# A Survey on Prevalence and Knowledge of Family Planning among Women of Childbearing Age in the Provincial Settings of the Gambia: A Descriptive Cross-Sectional Study

**DOI:** 10.1155/2020/8862290

**Published:** 2020-11-05

**Authors:** Amadou Barrow

**Affiliations:** Department of Public and Environmental Health, School of Medicine and Allied Health Sciences, University of the Gambia, Kanifing, Gambia

## Abstract

**Background:**

Family planning (FP) is one of the fundamental pillars of safe motherhood and reproductive health rights. In developing countries, women with unmet need for FP constitute a significant proportion of all women of reproductive age and it is an ongoing public health challenge in the Gambia. The study aimed to determine the women's proportion of contraceptive uptake and knowledge of FP methods.

**Methods:**

The study employed a community-based descriptive cross-sectional study conducted for 643 women of reproductive age (15–49 years) from the selected clusters in rural Gambia through a multistage sampling technique. A pretested structured interview questionnaire was used to collect data. Univariate analysis using frequencies and percentages were used to present results in this study. Data entry and analysis were done using IBM SPSS version 24.

**Results:**

The overall contraceptive prevalence rate was 30.4%, while the CPR for married or in the union was 34.2%. About 86% of women reported child spacing as the major benefits of FP, while 49.5% reported amenorrhea as the most common side effect of contraceptives. Injectable (Depo-Provera, Noristerat, and ) and pills (progesterone and combined) were the two most common FP methods used at 58.5% and 44.0%, respectively.

**Conclusion:**

The present study showed a moderately low contraceptive uptake. Thus, there is a need to focus FP services for women in rural areas, emphasizing the quality of services and gender equality. The study further recommends strengthening and mainstreaming of male involvement and religious leaders participation in FP interventions and the initiation of a communication program that explicitly promotes interspousal communication.

## 1. Introduction

In African countries including the Gambia, contraceptive uptake, fertility rates, and other reproductive health indicators in rural areas lag behind urban areas [1]. In 2019, there were about 1.9 billion women of childbearing age (15–49 years) worldwide. Globally, 1.1 billion need family planning; of these, 842 million are currently using contraceptives, while 270 million have an unmet need for contraception [2, 3]. The current global estimate for the need for family planning satisfied by modern methods based on Sustainable Development Goals (SDG) indicator 3.7.1 was 75.7% in 2019; yet less than half of the need for family planning was met in Middle and Western Africa [2]. As of 2010–2014, an estimated 36 abortions occur each year per 1000 women aged 15–44 in developing regions, compared with 27 in developed regions. As of now, there is no significant change in the estimates for developing regions [4]. Family planning (FP) offers some health benefits through the reduction of unintended (mistimed and unwanted) pregnancies [5]. These benefits include reduced spread of HIV to newborns; reduced maternal mortality and morbidity; reduced neonatal, infant, and child mortality [6]; reduced recourse of unsafe abortion; and improved education and employment opportunities for women (and men) who are able to delay the initiation of childbearing [5]. Thus, these the enhance progress towards achieving the goals in the 2030 Agenda for Sustainable Development, specifically, target 3.7, which supports universal access to reproductive health care, and target 5.6, which supports individuals' ability to exercise their reproductive rights [4].

In the Gambia, the national prevalence of modern contraceptive uptake among married or in union women is 16.3% while the utilization in urban areas is higher than in rural areas (17.7% versus 13.5%, respectively) [7]. The number of unintended pregnancies increased from 18,000 in 2012 to 20,000 in 2016 [8]. The consequences of an unwanted pregnancy include unsafe abortion practices that expose them to pelvic inflammatory diseases, ectopic pregnancy, secondary infertility, baby-dumping, STIs, including HIV/AIDS, and involvement in drug use and abuse [9]. Other specific individual benefits of FP include prevention of pregnancy-related risks and the unnecessary deaths of women [10, 11], reduction of infant mortality, prevention of STI, prevention of mother to child transmission of HIV [7, 12–14], prevention of adolescents from dropping out of school by reducing their pregnancies and enhancing the opportunity for girls getting an education [15], and improvement of economics of the family [16]. On the contrary, the number of pregnancies averted due to modern contraception methods increased from 8000 in 2013 to 10,000 in 2016 [8, 17]. The number of maternal deaths averted due to modern contraception methods increases from 40 in 2012 to 60 for 2016 [8]. Thus, FP was designed to create the opportunity of having the desired number of children with the required spacing of children desired [18].

Despite knowledge on FP's benefits and the attendant consequences of failure to adopt it as a birth control strategy, its usage rate is low [19]. Many factors have been adduced for the low contraceptive prevalence. Generally, the perception of people especially women in the developing countries towards FP deters them from using these services. A greater proportion of Africa's population resides in rural areas with attendant low contraceptive prevalence due to their fear of contraceptives and the side effects [20, 21]. Culture and traditions play a huge role in how people perceive FP innovations in developing nations and their behaviour tends to reflect the expectations of the influential custodians of these traditions [22]. FP's low usage also stemmed from the lack of general knowledge on contraceptives [23] and opposition from husbands [22]. Other factors responsible for low contraceptive prevalence have to do with background characteristics such as education, wealth, parity, religion, and place of residence [13, 24, 25]. In 2011, 19 cases of abandoned babies were registered by the Department of Social Welfare and this figure increased to 20 in 2012 and 25 in 2013 [26]. Misinformation about sex and lack of youth-friendly sexual and reproductive health services also pose challenges to young girls' sexual and reproductive health [9].

Thus, there are limited published studies on knowledge of rural women on FP in the Gambia. Therefore, this study aims to address this gap and explore reproductive age women's understanding of family planning in the context of four provincial LGAs of the Gambia. The results could be used to develop or inform policies and interventions to promote contraceptives' uptake by at-risk reproductive-age women in the Gambia.

## 2. Methods

A community-based descriptive cross-sectional study design using quantitative and qualitative research methods was conducted from December 2016 to January 2017 in provincial Gambia. The study area includes the four Local Government Areas (LGAs), namely, North Bank Region, Central River Region (North), Central River Region (South), and Upper River Region. The study targeted the women of reproductive age group (15–49 years old) across the four provincial LGAs in the Gambia.

### 2.1. Study Setting

The study was conducted in the Republic of the Gambia in four LGAs. The Gambia is situated on the western coast of Africa, and it is long and narrow in shape, extending 487 km into the hinterlands, with an average width of 24 km [27]. At the point where the River Gambia meets the Atlantic Ocean, the width of the country is twice the average at more than 48 km. The country is bound on three sides by Senegal and on the west by the Atlantic Ocean. The Gambia is one of the smallest states in West Africa with a land area of 10,689.28 km^2^, about one-tenth the size of Senegal [27]. The population of the Gambia is estimated to be 1.8 million [28]. The majority of the population are Muslims (95%), and there is a small proportion of Christians (4%) and followers of other indigenous religions [29].

The Basse LGA has it administrative headquarter at Basse with a population of 239,916 and a growth rate of 2.77%. It has a male population of 123,956 (51.7%) and female population of 115,960 (48.3%) [27]. The Kerewan LGA has its administrative headquarter in Kerewan, and it has a population of 221,054. It has a male population of 48.1% (104,931) and a female population of 51.8% (116,123) with a fertility rate of 6.3% [29]. The region has one tertiary Hospital, one major Health Centre, and 12 minor health centers [29]. The Janjanburreh LGA has its administrative headquarters at Janjanburreh, with a population of 126,910. It has a male population of 48.1% (60,001) and a female population of 51.9% (65,909) with a fertility rate of 7.0%. The region has no tertiary hospital and had one major health centre and four minor health centers. The main referral point for this region is Bansang Hospital [29]. Kuntaur LGA has its administrative headquarters at Kuntaur, with a population of 99,108. It has a male population of 47.7% (47,233) with a female population of 52.3% (51,875) with a fertility rate 7.2% [29].

#### 2.1.1. Measurement of Outcome Variables

The outcome variable was measured dichotomously (yes vs no) for current use of any FP methods while knowledge on FP was scored “1” for correct answer and “0” for wrong answers.

#### 2.1.2. Explanatory Variables

In this study, the independent variables for sociodemographic characteristics included age, place of residence, educational level, occupation, marital status, monthly income, parity, religion, and ethnicity. Other variables such as frequency of FP utilization and types of FP methods used were also obtained.

### 2.2. Eligibility Criteria

The study includes women of reproductive age (15–49 years old), married or unmarried and of any ethnic group or nationality, present during the study and consented to participate in the study. On the contrary, women who were unwilling to participate in the study, have any health/mental condition rendering it impossible to obtain an informed consent, reproductively incompetent due to hysterectomy, tubal ligation, etc. were excluded from the study.

### 2.3. Sample Size and Sampling Technique

The required sampling size was computed using single proportions [30] by considering the assumption of *Z*_∝_^2^ as the standard normal deviate, corresponding to 95% confidence level at which = 1.96 for a two-tailed test, *p* is the proportion in the target population estimated to have a particular characteristic (the unmet need for FP prevalence of 25% from the Gambia DHS 2013 [29]), and *d*^2^ is a degree of accuracy desired or maximum allowable difference from true proportion set at 5% (0.05) with a design effect of 2. The final sample size was 634 samples, which take into account a nonresponse rate of 10%. The study participants were selected using a multistage sampling technique that includes simple random sampling for selecting districts in a region, selecting Primary Health Care (PHC) circuits, selecting communities, and cluster sampling for selection of eligible participants in the communities.

### 2.4. Data Collection Procedure

Data were collected using a pretested interviewer-administered structured questionnaire. The questionnaire elicited information on their sociodemographic characteristics, use of FP, and knowledge towards FP methods. The interview was carried out in the major local languages based on the convenience of the respondents. The researcher was directly involved in data collection, cross-checking, data processing, and data analysis. Prior to the actual data collection, the tools were pretested on women (15–49) whose regions or districts were not chosen to be part of the actual study. Cronbach's Alpha test (0.89) was computed to measure reliability. Each filled questionnaire was rechecked just after the interview to correct wrong responses to assess appropriateness, content clarity, and comprehensiveness of the questions and time taken to fill out the questionnaire.

### 2.5. Data Analysis Plan

All data entry and analysis were performed using the Statistical Package for Social Sciences (SPSS) version 24. The data were examined using descriptive analysis, including means, percentages, and frequency distributions of study variables such as the sociodemographic variables, knowledge, and FP prevalence.

### 2.6. Ethical Considerations

Ethical clearance was obtained from the College of Medical Sciences' Research Ethics Committee, the University of Benin. Another ethical approval was obtained from both the Director of Health Services of the Ministry of Health and Social Welfare of the Gambia and the Scientific Committee of the University of the Gambia (RePubliC) and MRC/Gambia Government Joint Ethics Committee. Written informed consent was obtained from women (signed or thumb-printed), including parents of 15 to 17 years old. No penalty for withdrawal was implemented if the subjects (or their custodians) wish to withdraw from the study.

## 3. Results

A total of 634 women of childbearing age (15–49 years) in rural Gambia were recruited for the study with all of them participating, thus giving a response rate of 100%. Participants from Basse LGA constituted 220 (34.7%), Kerewan LGA 205 (32.3%), Janjanbureh LGA 117 (18.5%), and Kuntaur LGA 92 (14.5%).

### 3.1. Sociodemographic Characteristics

The mean age of the study participants was 26.2 years, with a standard deviation of ±6.7. One-hundred and ninety-five (30.8%) women were within the age group 20–24 years. From Table 1, most of the women (166 (26.2%)) had Arabic education, while only 25 (3.9%) had obtained the tertiary level of education. The majority of the women (230 (36.3%)) were exclusively housewives and the most represented ethnic group was Mandinka (296 (46.7%)). A large proportion of the participants were Muslims (620 (97.8%)), and 564 (89.0%) were married. More than half of the participants earned less than or equal to D2000 in a month.

### 3.2. Knowledge of Family Planning Methods

A large proportion of the participants (430 (89.4%)) knew about pills (progesterone only and combined), 405 (84.2%) knew about injectables (Depo-Provera, Noristerat, and Norigynon), while 175 (36.4%) knew about implants (implanon and jadelle). As for the barrier methods, 75 (15.6%) knew about female condom while about 21 (4.4%) mentioned IUD as shown in Table 2. Concerning the natural methods, prolonged breastfeeding accounted for 72 (15.0%) and rhythm method (safe period) (33 (6.9%)). The majority of the participants (369 (82.6%)) know how to use pills (progesterone and combined). A considerable proportion of the participants (31.1%) reported knowledge of the use of implants (implanon and jadelle) as compared to the coitus interruptus (withdrawal), which accounted for 11 (2.5%).

Nine in every ten women reported that contraceptives are beneficial. Out these, 368 (85.6%) reported child spacing, followed by prevention of unwanted pregnancy 257 (59.8%), limiting family size 135 (31.4%), and enhancement/improvement of family economic status 33 (17.0%) as the benefits of using contraceptives as shown in Table 3. Slightly more than half of the participants reported that contraceptives are harmful or have side effects. Amenorrhea and secondary infertility accounted for 139 (49.5%) and 88 (31.3%), respectively.

### 3.3. Prevalence and Types of Contraceptives Used by Women

In terms of specific FP prevalence by methods, slightly more than half (58.5%) reported currently using injectables (Depo-Provera, Noristerat, and Norigynon), followed by pills (progesterone and combined) at 44.0%, as shown in Table 4. Among the current FP users, 175 (90.7%) reported that child spacing and 102 (52.8%) reported unwanted pregnancy as their reasons for FP use. Almost half of the participants reported having used FP before. Out of the 341 (53.8%) women who never used FP before, 137 (40.7%) and 122 (36.2%) reported fear of side effects and preference for a male child as their primary reasons for not using FP, respectively. However, other reasons such as religious beliefs 101 (30.0%), partner refusal 83 (24.6%), and lack of knowledge on how to use it 56 (16.6%) were revealed by the study.

As shown in Table 5, Basse LGA recorded the highest for both the number of injectable users (Depo-Provera, Noristerat, and Norigynon) and pills (progesterone and combined) at 36 (69.2%) and 24 (46.2%). Regarding traditional methods, Kuntaur recorded the highest 4 (9.3%), while Kerewan LGA recorded the lowest 2 (3.5%). Of the 111 (58.5%) who were currently using injectable (Depo-Provera, Noristerat, and Norigynon), 30 (69.8%) were within 20–24 years of age. In the area of religion, 109 (57.7%) among Muslims were reported to be using injectables (Depo-Provera, Noristerat, and Norigynon). A total of 71 (58.2%) women with parity of 0–4 were using injectables (Depo-Provera, Noristerat, and Norigynon) at the study time. Slightly more than half of women with either no educational background or with Arabic education were reported to be currently using pills (progesterone and combined). The majority of women 42 (58.3%) that were exclusively housewives reported to be using injectables (Depo-Provera, Noristerat, and Norigynon) and 29 (40.3%) for pills (progesterone and combined). Among those women who earned more than D4000 a month, 32 (69.6%) were primarily using injectables (Depo-Provera, Noristerat, and Norigynon).

## 4. Discussion

Participants' mean age was similar to that in the studies conducted in 2017 among women of childbearing age in a rural community of Southern Nigeria [31] and Oyo State, Nigeria [32]. The larger proportion of women were found within their peak age of reproduction. Two-thirds of the women have some education forms up to the secondary level, similar to that of a study done in Osun State [33]. Women with no formal education were found to be high non-FP users, which could have accounted for the parity seen among women. It has also been demonstrated that the higher the educational status, the less likely the women's parity. Male preference for the couple is also an important contributory factor for high parity, which is not within this study's scope. Similar findings were reported in a study conducted in Edo State, Nigeria, and also in Ikeji Arakeyi, Osun State, Nigeria, where the higher proportion of participants were married [32, 34].

Since most of the rural Gambia women knew about pills and injectables, this might be due to the availability and acceptability of these methods by the community. In this study, the majority of rural residents had little knowledge about other types of contraceptive methods, especially long-term and permanent methods hence less utilization of other methods. Rural areas in the Gambia are generally patriarchal in nature, and low literacy rate and deep-rooted cultural practices could contribute to the low utilization of Implanon and IUD. The majority of the decisions in such settings, especially family planning, are taken by husbands/partners due to women's economic dependence, low educational level, and existing culture. Similar findings have been reported from Honduras where urban women were more likely to decide on modern contraceptive use than rural women [35]. These disparities could also explain the national variations between rural and urban areas regarding the contraceptive prevalence and knowledge gap on FP methods and commodities. Thus, the role of media in various forms to raised awareness remains critical for rural communities instead of urban dwellers.

The study revealed that high awareness level on FP methods and these commodities' availability does not substantially translate into widespread behavior changes among women in rural settings. However, there were unusual findings on the women's reaction to FP services, which may be attributed to the higher proportion of women with advanced age. It is critical to address these persistent challenges with rural men and women alike to promote mutual comprehension, awareness, and decision-making regarding the use of modern contraceptives.

The overall contraceptive prevalence and that of married/in union women are higher than both the country's 2013 DHS [36] and 2018 MICS [7] at 7.1% and 16.3%, respectively. However, the mCPR was lower than the findings of Kenya's 2009 DHS [37] and 2014 DHS [38] at 53.6% and 58.0% of currently married women were using some contraception methods, respectively. In terms of knowledge on the available FP methods, the most common modern contraceptive methods mentioned were pills and injectables. These could be due to the expansion or decentralization of RCH programs across the country's length and breadth, including the rural communities. Additionally, there have been intensive FP promotion interventions in the country by Gambia Family Planning Association, Peer Health Education, which may have triggered increased demand for FP services.

Provincial women pointed out that their male partner's resistance to FP was a major barrier to use, even in the rare cases of joint decision-making [39]. Although men play the decision maker's role, they are often detached from and lack interest in reproductive health issues, particularly in rural patriarchal communities [40]. A study in rural Ghana noted that although most women considered FP acceptable, a higher percentage of women expressed that they would require their partners' permission before adopting a modern method [41]. Consistent with this study's results, this finding suggests that a woman's conviction is insufficient to ensure the actual uptake of modern contraceptive methods. However, the Ghanaian study also showed that men and women had similar levels of acceptance of FP, which could point to inadequate spousal communication leading to an inaccurate perception of male partners' opinions in some instances [41].

## 5. Policy Implication

These findings have some programmatic implications. Many of the women who were nonusers and open to adopting FP methods in the future as a way to limit births having reached their desired number of children. However, participants also cited inadequate spousal communication, limited knowledge, and fear of side effects and cultural norms as additional factors in their decision as to whether or not to eventually adopt a modern contraceptive method, even after reaching their ideal family size. This study creates the need for women empowerment through income-generation activities and improving the whole community's living standards. Advocating women education and increasing enrollment in both primary and secondary schools and eventually at University level might help solve the income (wealth index) problem and increase awareness on FP methods and eventually increase contraceptive use.

## 6. Study Limitations

Like any other study, our population of interest was women of childbearing age and could be generalizable in similar rural settings in the Gambia. Since the study was a descriptive cross-sectional study design, it can only help formulate a hypothesis and not determine causality between variables under study. It did not extend to study the determinants of unmet need for FP, total demand for FP, health system factors' influence on both unmet needs for FP, and its uptake. The information obtained was based on self-reporting, and the authenticity of the claim may not be readily confirmable. Furthermore, minimal analytical tests were done in this paper. More advanced analytical statistical tests such as regression analysis should identify predictors of FP uptake in rural communities. Male perspectives towards FP were not explored in this paper.

## 7. Conclusion

The gap between FP services and the current utilization could be bridged by increasing FP commodities' accessibility and acceptability to reduce rural Gambia's fertility level. Therefore, the population-based programme should motivate both nonusers and “dropouts” to resume practicing contraception to prioritize providing appropriate information, education and communication measures, and improved supervision at the community level. The programme certainly needs to give due consideration in improving the quality of care being offered to acceptors. This issue can be better addressed under the current RCH service delivery strategy of providing services from static clinics, attended by village health workers in addition to community health workers and facility-based healthcare service providers. The FP providers need to be given adequate training on counseling, screening, and management of side effects. Spousal communication between in union/married partners on FP matters is an essential intermediate step along the path to their eventual adoption and FP methods' sustained use. Future studies should also attempt to assess the factors that determine the unmet need for FP among women in urban areas of the Gambia. The significance of such studies will inform the place of residence specific programmatic actions. Moreover, such studies will provide adequate information to offer a solution to the currently increasing fertility among women in poor urban settlements and rural areas.

## Figures and Tables

**Table 1:** Sociodemographic characteristics of participants.

Variables	Frequency (*n* = 634)	Percent
Local government areas		
Basse	220	34.7
Kerewan	205	32.3
Janjanbureh	117	18.5
Kuntaur	92	14.5

Age of women (years)		
15–19	93	14.7
20–24	195	30.8
25–29	177	27.9
30–34	80	12.6
35–39	54	8.5
40–44	30	4.7
45–49	5	0.8
Mean age (standard deviation): 26.2 (6.7) years		

Educational status		
Never attended school	154	24.3
Arabic education	166	26.2
Primary education	122	19.2
Secondary education	167	26.3
Tertiary education	25	3.9

Occupational status		
Exclusively housewife	230	36.3
Farming	165	26
Trading	114	18
Civil servant	44	6.9
Student	44	6.9
Self-employed	27	4.3
Muslim religious teacher	10	1.6

Religion		
Muslim	620	97.8
Christian	10	1.6
Traditional African religion	4	0.6

Marital status		
Married/in union	564	89.0
Single	70	11.0

Monthly income		
Less than or equal to D2000	360	56.8
D2001–D4000	158	24.9
More than D4000	116	18.3

Ethnicity		
Mandinka	296	46.7
Fula	152	24
Sarahule	81	12.8
Wollof	61	9.6
Serere	28	4.4
Aku	15	2.4
Manjago	1	0.2

**Table 2:** Knowledge on contraceptive methods and how to use them.

Variables	Know about it		Know how to use it	
	Frequency (*n* = 481)	Percent	Frequency (*n* = 447)	Percent
FP/Contraceptive methods^*∗*^				
Pills				
Pills (progesterone only and combined)	430	89.4	369	82.6
Emergency contraceptives	13	2.7	11	2.5

Injectables				
Injectable (Depo-Provera, Noristerat, and Norigynon)	405	84.2	352	78.7

Barrier				
Condom	75	15.6	57	12.8
Intrautrine device (IUD)	21	4.4	19	4.3
Diaphragm	10	2.1	9	2.0
Suppositories, e.g., foam tablets	16	3.3	11	2.5

Surgery				
Female sterilization	11	2.3	6	1.3

Implants				
Implants (implanol and jadelle)	175	36.4	139	31.1

Natural method				
Rhythm method (safe period)	33	6.9	27	6.0
Coitus interruptus (withdrawal)	11	2.3	11	2.5
Periodic abstinence	23	4.8	14	3.1
Prolonged breastfeeding	72	15.0	63	14.1
Douching	7	1.5	6	1.3

Others				
Traditional methods	68	14.1	49	11.0

^*∗*^Multiple responses.

**Table 3:** Knowledge of benefits and side effects of contraceptives.

Variables	Frequency	Percent
Contraceptives are beneficial (*n* = 481)		
Yes	430	89.4
No	51	10.6

Benefits of contraceptives^*∗*^ (*n* = 430)		
Child spacing	368	85.6
Prevention of unwanted pregnancy	257	59.8
Limit family size	135	31.4
Prevention of STIs	77	17.9
Enhance/improve family economic status	73	17.0
Increase sexual pleasure	29	6.7
Reduce overpopulation	1	0.2

Contraceptives are harmful or have side effects (*n* = 481)		
Yes	283	58.8
No	190	39.5
I do not know	8	1.7

Harmful/side effects of contraceptives^*∗*^ (*n* = 283)		
Amenorrhea	139	49.5
Irregular menses	118	42.0
Weight gain	109	38.8
Heavy menses	107	38.1
Secondary infertility	88	31.3
Weight loss	34	12.1
Extramarital affairs	23	8.2
Condom burst/spillage	11	3.9
None of the above	7	2.5

Ways of avoiding pregnancy^*∗*^ (*n* = 481)		
Use of modern contraceptives	455	94.6
Prolonged breastfeeding (LAM)	207	43.0
Withdrawal	49	10.2
Avoid coitus (sex)	38	7.9
Rhythm methods (during safe period)	36	7.5
Traditional methods	24	5.0
Douching	3	0.6

^*∗*^Multiple responses.

**Table 4:** Prevalence of FP uptake among the participants.

Variables	Frequency	Percent
Women currently using FP		
Yes	193	30.4
No	441	69.6

FP methods currently using^*∗*^ (*n* = 193)		
Injectable (Depo-Provera, Noristerat, and Norigynon)	113	58.5
Pills (progesterone and combined)	85	44.0
Implants (implanon and jadelle)	20	10.4
Prolonged breastfeeding	15	7.8
Traditional methods	13	6.7
Rhythm method (safe period)	8	4.1
Female condom	6	3.1
Suppositories, e.g., foam tablets	1	0.5
Emergency contraceptives	1	0.5
Female sterilization	1	0.5
Coitus interruptus (withdrawal)	1	0.5
Periodic abstinence	1	0.5

Reasons for using FP^*∗*^ (*n* = 193)		
Child spacing	175	90.7
Unwanted pregnancy	102	52.8
Affordable and available	68	35.2
Suitable and reliable	42	21.8
No need for more children	41	21.2
Little or no side effects	21	10.9
No reason	3	1.6
Following my partner's decision	3	1.6

Ever used FP before		
Yes	293	46.2
No	341	53.8

FP methods used before^*∗*^ (*n* = 293)		
Injectable (Depo-Provera, Noristerat, and Norigynon)	202	68.9
Pills (progesterone and combined)	170	58.0
Implants (implanon and jadelle)	37	12.6
Prolonged breastfeeding	33	11.3
Condom (female)	16	5.5
Traditional methods	16	5.5
Rhythm method (safe period)	13	4.4
Suppositories, e.g., foam tablets	2	0.7
Emergency contraceptives	1	0.3
Diaphragm	1	0.3
Intrauterine device (IUD)	1	0.3
Douching	1	0.3
Periodic abstinence	1	0.3

Reasons for not using FP before^*∗*^ (*n* = 341)		
Fear of side effects	137	40.7
Preference for male child	122	36.2
It is against my religious beliefs	101	30.0
Partner refusal or disapproval	83	24.6
Do not know how to use it	56	16.6
Do not know where to access them	28	8.3
Reduce sexual pleasure	13	3.9
I cannot always afford the cost	12	3.6

^*∗*^Multiple responses.

**Table 5:** Prevalence of FP currently used by sociodemographic variables.

Variables	FP methods being used currently (*n* = 193)^*∗*^												
	Female condom	Suppositories	Emer. Cp	Inject.	Female ster.	Rhythm method	Withdrawal	Pills	Impl.	Periodic abst.	Prolonged bf	T. methods	Total *n* (%)
Local government area													
Kerewan	0 (0.0)	1 (1.8)	0 (0.0)	28 (49.1)	0 (0.0)	5 (8.5)	0 (0.0)	26 (45.6)	8 (14.0)	1 (1.8)	4 (7.0)	2 (3.5)	57 (29.5)
Kuntaur	5 (11.6)	0 (0.0)	1 (2.3)	26 (60.5)	1 (2.3)	3 (7.0)	1 (2.3)	18 (41.9)	5 (11.6)	0 (0.0)	6 (14.0)	4 (9.3)	43 (22.3)
Janjanbureh	0 (0.0)	0 (0.0)	0 (0.0)	23 (56.1)	0 (0.0)	0 (0.0)	0 (0.0)	17 (41.5)	7 (17.1)	0 (0.0)	0 (0.0)	3 (7.3)	41 (21.2)
Basse	1 (1.9)	0 (0.0)	0 (0.0)	36 (69.2)	0 (0.0)	0 (0.0)	0 (0.0)	24 (46.2)	0 (0.0)	0 (0.0)	5 (9.6)	4 (7.7)	52 (26.9)

Age group of participants													
15–19	0 (0.0)	0 (0.0)	0 (0.0)	5 (50.0)	0 (0.0)	1 (10.0)	0 (0.0)	5 (50.0)	1 (10.0)	0 (0.0)	0 (0.0)	0 (0.0)	10 (5.2)
20–24	2 (4.7)	0 (0.0)	0 (0.0)	30 (69.8)	0 (0.0)	2 (4.7)	0 (0.0)	13 (30.2)	4 (9.3)	0 (0.0)	4 (9.3)	3 (7.0)	43 (22.3)
25–29	2 (3.3)	1 (1.7)	1 (1.7)	30 (30.0)	0 (0.0)	0 (0.0)	0 (0.0)	31 (51.7)	8 (13.3)	1 (1.7)	5 (8.3)	3 (5.0)	60 (31.1)
30–34	0 (0.0)	0 (0.0)	0 (0.0)	22 (61.1)	0 (0.0)	2 (5.6)	0 (0.0)	17 (47.2)	3 (8.3)	0 (0.0)	4 (11.1)	2 (5.6)	36 (18.7)
35–39	1 (3.4)	0 (0.0)	0 (0.0)	20 (69.0)	0 (0.0)	2 (6.9)	0 (0.0)	12 (41.4)	1 (3.4)	0 (0.0)	2 (6.9)	1 (3.4)	29 (15.0)
40–44	1 (7.7)	0 (0.0)	0 (0.0)	4 (30.8)	1 (7.7)	1 (7.7)	1 (7.7)	7 (53.8)	3 (23.1)	0 (0.0)	0 (0.0)	2 (15.4)	13 (6.7)
45–49	0 (0.0)	0 (0.0)	0 (0.0)	2 (50.0)	0 (0.0)	0 (0.0)	0 (0.0)	0 (0.0)	0 (0.0)	0 (0.0)	0 (0.0)	2 (50.0)	2 (1.0)

Ethnicity													
Mandinka	4 (4.4)	0 (0.0)	0 (0.0)	51 (56.7)	0 (0.0)	5 (5.6)	0 (0.0)	37 (41.1)	12 (13.3)	1 (1.1)	7 (7.8)	9 (10.0)	90 (46.6)
Fula	0 (0.0)	1 (2.1)	0 (0.0)	33 (68.8)	1 (2.1)	1 (2.1)	1 (2.1)	18 (37.5)	3 (6.3)	0 (0.0)	2 (4.2)	2 (4.2)	48 (24.9)
Sarahule	0 (0.0)	0 (0.0)	0 (0.0)	9 (56.3)	0 (0.0)	0 (0.0)	0 (0.0)	10 (62.5)	0 (0.0)	0 (0.0)	0 (0.0)	0 (0.0)	16 (8.3)
Wollof	1 (4.5)	0 (0.0)	1 (4.5)	10 (45.5)	0 (0.0)	1 (4.5)	0 (0.0)	14 (63.6)	3 (13.6)	0 (0.0)	2 (9.1)	1 (4.5)	22 (11.4)
Serere	1 (9.1)	0 (0.0)	0 (0.0)	5 (45.5)	0 (0.0)	1 (9.1)	0 (0.0)	5 (45.5)	2 (18.2)	0 (0.0)	4 (36.4)	1 (9.1)	11 (5.7)
Aku	0 (0.0)	0 (0.0)	0 (0.0)	5 (83.3)	0 (0.0)	0 (0.0)	0 (0.0)	1 (16.7)	0 (0.0)	0 (0.0)	0 (0.0)	0 (0.0)	6 (3.1)

Religion													
Muslim	5 (2.6)	1 (0.5)	1 (0.5)	109 (57.7)	1 (0.5)	8 (4.2)	1 (0.5)	85 (45.0)	20 (10.6)	1 (0.5)	15 (7.9)	13 (6.9)	189 (97.9)
Christian	1 (25.0)	0 (0.0)	0 (0.0)	4 (100.0)	0 (0.0)	0 (0.0)	0 (0.0)	0 (0.0)	0 (0.0)	0 (0.0)	0 (0.0)	0 (0.0)	4 (2.1)

Marital status													
Married/in union	4 (2.3)	0 (0.0)	1 (0.6)	108 (61.7)	0 (0.0)	6 (3.4)	0 (0.0)	76 (43.4)	17 (9.7)	1 (0.6)	15 (8.6)	11 (6.3)	175 (90.7)
Single	2 (11.1)	1 (5.6)	0 (0.0)	5 (27.8)	1 (5.6)	2 (11.1)	1 (5.6)	9 (50.0)	3 (16.7)	0 (0.0)	0 (0.0)	2 (11.1)	18 (9.3)

Parity													
0–4	5 (4.1)	0 (0.0)	0 (0.0)	71 (58.2)	1 (0.8)	4 (3.3)	1 (0.8)	51 (41.8)	10 (8.2)	1 (0.8)	8 (6.6)	6 (4.9)	122 (65.2)
5–8	1 (1.8)	0 (0.0)	1 (1.8)	34 (60.7)	0 (0.0)	3 (5.4)	0 (0.0)	28 (50.0)	7 (12.5)	0 (0.0)	6 (10.7)	5 (8.9)	56 (29.9)
9+	0 (0.0)	0 (0.0)	0 (0.0)	7 (77.8)	0 (0.0)	0 (0.0)	0 (0.0)	2 (22.2)	2 (22.2)	0 (0.0)	1 (11.1)	2 (22.2)	9 (4.8)

Level of education													
Never attended school	1 (2.6)	0 (0.0)	0 (0.0)	22 (56.4)	0 (0.0)	2 (5.1)	0 (0.0)	17 (43.6)	3 (7.7)	0 (0.0)	0 (0.0)	3 (7.7)	39 (20.2)
Arabic edu.	0 (0.0)	0 (0.0)	0 (0.0)	24 (61.5)	0 (0.0)	0 (0.0)	0 (0.0)	19 (48.7)	3 (7.7)	0 (0.0)	5 (12.8)	4 (10.3)	39 (20.2)
Primary	1 (2.0)	1 (2.0)	1 (2.0)	30 (61.2)	1 (2.0)	2 (4.1)	1 (2.0)	18 (36.7)	6 (12.2)	0 (0.0)	2 (4.1)	3 (6.1)	49 (25.4)
Secondary	4 (7.0)	0 (0.0)	0 (0.0)	32 (56.1)	0 (0.0)	4 (7.0)	0 (0.0)	28 (49.1)	6 (10.5)	1 (1.8)	8 (14.0)	3 (5.3)	57 (29.5)
Tertiary	0 (0.0)	0 (0.0)	0 (0.0)	5 (55.6)	0 (0.0)	0 (0.0)	0 (0.0)	3 (33.3)	2 (22.2)	0 (0.0)	0 (0.0)	0 (0.0)	9 (4.7)

Occupation of the participants													
Ex. housewife	1 (1.4)	0 (0.0)	1 (1.4)	42 (58.3)	0 (0.0)	3 (4.2)	0 (0.0)	29 (40.3)	8 (11.1)	1 (1.4)	3 (4.2)	3 (4.2)	72 (37.3)
Self-employed	0 (0.0)	0 (0.0)	0 (0.0)	5 (50.0)	0 (0.0)	0 (0.0)	0 (0.0)	6 (60.0)	2 (20.0)	0 (0.0)	0 (0.0)	2 (20.0)	10 (5.2)
Farming	2 (7.1)	0 (0.0)	0 (0.0)	8 (28.6)	1 (3.6)	3 (10.7)	1 (3.6)	16 (57.1)	4 (14.3)	0 (0.0)	1 (3.6)	2 (7.1)	28 (14.5)
Student	0 (0.0)	0 (0.0)	0 (0.0)	2 (50.0)	0 (0.0)	1 (25.0)	0 (0.0)	2 (50.0)	1 (25.0)	0 (0.0)	0 (0.0)	0 (0.0)	4 (2.1)
Trading	1 (1.8)	1 (1.8)	0 (0.0)	43 (75.4)	0 (0.0)	1 (1.8)	0 (0.0)	22 (38.6)	4 (4.0)	0 (0.0)	10 (17.5)	6 (10.5)	57 (29.5)
Civil servant	2 (9.5)	0 (0.0)	0 (0.0)	12 (57.1)	0 (0.0)	0 (0.0)	0 (0.0)	10 (47.6)	1 (4.8)	0 (0.0)	1 (4.8)	0 (0.0)	21 (10.9)
Muslim religious teacher	0 (0.0)	0 (0.0)	0 (0.0)	1 (100.0)	0 (0.0)	0 (0.0)	0 (0.0)	0 (0.0)	0 (0.0)	0 (0.0)	0 (0.0)	0 (0.0)	1 (0.5)

Average monthly income													
≤D2000	1 (1.1)	1 (1.1)	1 (1.1)	41 (46.6)	1 (1.1)	4 (4.5)	1 (1.1)	43 (48.9)	12 (13.6)	1 (1.1)	2 (2.3)	4 (4.5)	88 (46.6)
D2001–D4000	1 (1.8)	0 (0.0)	0 (0.0)	37 (67.3)	0 (0.0)	3 (5.5)	0 (0.0)	22 (40.0)	5 (9.1)	0 (0.0)	6 (10.9)	7 (12.7)	55 (29.1)
>D4000	3 (6.5)	0 (0.0)	0 (0.0)	32 (69.6)	0 (0.0)	1 (2.2)	0 (0.0)	18 (39.1)	3 (6.5)	0 (0.0)	6 (13.0)	2 (4.3)	46 (24.3)

^*∗*^Multiple response.

## Data Availability

The datasets are available on reasonable request from the corresponding author.

## Abbreviations

CPR: Contraceptive prevalence rate
GBoS: Gambia Bureau of Statistics
HIV: Human immunodeficiency virus
IEC: Information, education, and communication
LGA: Local government area
mCPR: Modern contraceptive prevalence rate
MOHERST: Ministry of Higher Education, Research, Science and Technology
SPSS: Statistical package for social sciences
USAID: US Agency for International Aid
UNICEF: United Nations Children's Fund
UNDP: United Nations Development Program
UNFPA: United Nations Population Fund
WHO: The World Health Organization.
